# A survey of the perceptions of barriers to and facilitators of cardiac rehabilitation in healthcare providers and policy stakeholders

**DOI:** 10.1186/s12913-022-08298-3

**Published:** 2022-08-05

**Authors:** Chul Kim, Hae-Bin Kwak, Jidong Sung, Jae-Young Han, Jang Woo Lee, Jong Hwa Lee, Won-Seok Kim, Heui Je Bang, Sora Baek, Kyung Lim Joa, Ae Ryoung Kim, So Young Lee, Jihee Kim, Chung Reen Kim, Oh. Pum Kwon, Min Kyun Sohn, Chang-Won Moon, Jae-In Lee, Sungju Jee

**Affiliations:** 1 Department of Rehabilitation Medicine, Inje University Sanggye Paik Hospital, Seoul, South Korea; 2 Department of Rehabilitation Medicine, Chungnam National University Hospital, Chungnam National Univeristy College of Medicine, Daejeon, South Korea; 3 Division of Cardiology, Department of Medicine, Sungkyunkwan University School of Medicine, Seoul, South Korea; 4 Department of Physical Medicine and Rehabilitation, Chonnam National University Hospital, Chonnam National University Medical School, Gwangju, South Korea; 5 Department of Physical Medicine and Rehabilitation, National Health Insurance Service Ilsan Hospital, Goyang, South Korea; 6 Department of Physical Medicine and Rehabilitation, Dong-A University College of Medicine, Busan, South Korea; 7 Department of Rehabilitation Medicine, Seoul National University College of Medicine, Seoul National University Bundang Hospital, Seongnam, South Korea; 8 Department of Rehabilitation Medicine, Chungbuk National University, College of Medicine, Cheongju, South Korea; 9 Department of Rehabilitation Medicine, Kangwon National University School of Medicine, Chuncheon, South Korea; 10 Department of Physical Medicine and Rehabilitation, Inha University Hospital, Incheon, South Korea; 11 Department of Rehabilitation Medicine, Kyungpook National University Hospital, Kyungpook National University School of Medicine, Daegu, South Korea; 12 Department of Rehabilitation Medicine, Jeju National University Hospital, Jeju National University College of Medicine, Jeju, South Korea; 13 Department of Rehabilitation Medicine, Wonkwang University Hospital, Wonkwang University Medical School, Iksan, South Korea; 14 Department of Physical Medicine and Rehabilitation, Ulsan University Hospital, University of Ulsan College of Medicine, Ulsan, South Korea

**Keywords:** Administrative personnel, Awareness, Cardiac rehabilitation, Cardiac infarction, Government stakeholder, Health personnel, Hospital administrator

## Abstract

**Background:**

Cardiac rehabilitation (CR) is a prognostic management strategy to help patients with CVD achieve a good quality of life and lower the rates of recurrence, readmission, and premature death from disease. Globally, cardiac rehabilitation is poorly established in hospitals and communities. Hence, this study aimed to investigate the discrepancies in the perceptions of the need for CR programs and relevant health policies between directors of hospitals and health policy personnel in South Korea to shed light on the status and to establish practically superior and effective strategies to promote CR in South Korea.

**Methods:**

We sent a questionnaire to 592 public health policy managers and directors of selected hospitals, 132 of whom returned a completed questionnaire (response rate: 22.3%). The participants were categorized into five types of organizations depending on their practice of PCI (Percutaneous Coronary Intervention), establishment of cardiac rehabilitation, director of hospital, and government's policy makers. Differences in the opinions between directors of hospitals that perform/do not perform PCI, directors of hospitals with/without cardiac rehabilitation, and between hospital directors and health policy makers were analyzed.

**Results:**

Responses about targeting diseases for cardiac rehabilitation, patients’ roles in cardiac rehabilitation, hospitals’ roles in cardiac rehabilitation, and governmental health policies’ roles in cardiac rehabilitation were more positive among hospitals that perform PCI than those that do not. Responses to questions about the effectiveness of cardiac rehabilitation and hospitals’ roles in cardiac rehabilitation tended to be more positive in hospitals with cardiac rehabilitation than in those without. Hospital directors responded more positively to questions about targeting diseases for cardiac rehabilitation and governmental health policies’ roles in cardiac rehabilitation than policy makers, and both hospitals and public organizations provided negative responses to the question about patients’ roles in cardiac rehabilitation. Responses to questions about targeting diseases for cardiac rehabilitation, patients’ roles in cardiac rehabilitation, and governmental health policies’ roles in cardiac rehabilitation were more positive in hospitals that perform PCI than those that do not and public organizations.

**Conclusions:**

Hospitals must ensure timely referral, provide education, and promote the need for cardiac rehabilitation. In addition, governmental socioeconomic support is needed in a varity of aspects.

**Supplementary Information:**

The online version contains supplementary material available at 10.1186/s12913-022-08298-3.

## Background

Cardiovascular disease (CVD) is a chronic condition that requires acute phase treatment in the hospital and lifelong self-care. CVD mortality in Korea rose by 42.8% over the past 10 years. It has become the second-leading cause of death in Korea since 2014 [1]. According to a 2019 Statistics Korea report, 60.4 per 100,000 population died from CVD, with ischemic heart disease (e.g., myocardial infarction and angina) accounting for 26.7% and other heart diseases (e.g., heart failure and valvular disease) accounting for 33.8% [2].

Cardiac rehabilitation (CR) is a prognostic management strategy to help patients with CVD achieve a good quality of life and lower the rates of recurrence, readmission, and premature death from disease [3]. Clinical practice guidelines for cardiac rehabilitation strongly recommend CR with a high level of evidence. A Taiwanese study on Asians that compared the insurance claims data until the year 2008 between 442 patients (15%) with and 2396 (84.4%) without CR after undergoing their first percutaneous coronary intervention (PCI) during 2000–2007 showed that 69 (15.6%) patients in the CR group and 840 (35.1%) in the non-CR group ended up undergoing hybrid coronary revascularization. The risk of undergoing another revascularization during the follow-up period was substantially low (0.48) in the CR group [4].

However, the actual participation rate of CR among patients who require it is still low even in medically advanced countries (approximately 30–40%) due to numerous barriers [5]. The availability of CR was 68% in high-income countries, but it was significantly lower at 8.3% in low-income countries, and only 38.8% globally were available [6]. A survey of the current status of CR in South Korean cardiocerebrovascular centers showed that approximately 47% of patients who received inpatient care for acute myocardial infarction underwent CR after discharge, with 36% undergoing early assessment and 17% continuing CR. However, the rates varied widely across facilities and compared with the high CR referral rate, CR participation and completion rates were relatively low, in Korea [7]. Worldwide, CR available is only half, and participation is restricted because most patients cannot be accepted even if they are provided [8].

Reasons for low CR participation include distance, work responsibilities, lack of time, transportation problems, and comorbidities [9]. A South Korean study showed that travel-related difficulties, lack of time, cost burden, and inadequate motivation were associated with low CR participation [10]. Moreover, there are inadequate CR facilities in South Korean hospitals and communities, and self-care, including lifestyle modification for long-term management, is not appropriately incorporated. Additionally, an effective delivery system linking discharged patients to community-based management or a systematic strategy to improve patients’ hospital visits and medication adherence is also lacking.

Hence, this study aimed to investigate the discrepancies in the perceptions of the need for CR programs and relevant health policies between directors of hospitals and health policy personnel in South Korea to shed light on the status and to establish practically superior and effective strategies to promote CR in South Korea.

## Methods

### Study population

A questionnaire was sent to the directors of hospitals who can influence CR programs’ implementation and operation and those of relevant divisions at the National Evidence-based Healthcare Collaborating Agency, National Rehabilitation Center, and Health Insurance Review and Assessment as health policy personnel.

Organizations not suitable per the objective and purpose of this research project or those not willing to participate in the project were excluded, and regional cardiocerebrovascular centers nationwide, university hospitals in the Seoul Metropolitan area, and National Health Insurance Service Ilsan Hospital participated in this multicenter study.

Data were collected from July 6, 2020 to December 31, 2020.

### Instruments

The questionnaire for hospital managers-policy personnel was developed by collecting and analyzing Korean and international CR clinical practice guidelines, and other relevant study data. The questionnaire was developed to meet the domestic situation by referring to the CR Barriers Scale and CR Referral Tools of York University, Canada [11–13] and Validated CR Scales of the International Council of Cardiovascular Prevention and Rehabilitation [9]. The questionnaire comprised one short-answer item and 15 multiple-choice items about effectiveness (Q1–3), target diseases (Q4), patients’ (Q5) and hospitals’ roles (Q6–9), and those of governmental health policies in CR (Q10–15). Each item was rated on a scale of 1–5 (strongly disagree to strongly agree), where scores of 1–2, 3, and 4–5 points indicated negative, neutral, and positive perceptions, respectively. A higher score indicates stronger agreement with the associated statement. The questionnaire is attached as an additional file [Additional file 1].

### Analysis

The response rate was calculated as the percentage of participants who submitted the completed questionnaire within the given deadline. The participating organizations were divided into five types depending on whether they performed PCI and had a CR system and whether the respondent was a hospital director or a civil servant. The data were non-normally distributed; thus, nonparametric tests were used. The responses for each item were compared between organizations using the Kruskal-Wallis test. Statistics are presented as medians and quartiles 1 to 3. *P*-values < 0.05 indicated statistical significance.

The participants were reclassified according to their organizations’ characteristics to compare the responses between directors of hospitals that did and did not perform PCI, between directors of hospitals with and without CR, and between hospital directors and health policy directors using the Mann-Whitney test. The discrepancies in the opinions among directors of hospitals that performed PCI, directors of hospitals that did not perform PCI, and health policy directors were analyzed using the Kruskal-Wallis test. For statements that significantly differed among the respondents, type 1 error was adjusted for using the Bonferroni test, and groups were paired in two and analyzed with the Mann-Whitney test for post hoc comparison (statistical significance *p* < 0.017). The data were analyzed using SPSS 20.0 software. This study was approved by each organizations’ Institutional Review Board (CNUH-IRB no. 2020–05-059).

## Results

### Participant characteristics and response rate

The questionnaire was sent to 592 participants, namely 31 directors of hospitals that performed PCI and CR, 13 directors of hospitals with regional centers, 117 directors of hospitals that performed PCI but not CR, 408 directors of hospitals that did not perform PCI, and 23 civil servants belonging to public agencies. A total of 132 participants submitted the completed questionnaires, with a response rate of 22.30% (Table 1).

**Table 1:** Survey candidate and response

	Candidate	Response	Response rate (%)
PCI, CR	31	13	41.94
CCRC	13	4	30.77
PCI, Non-CR	117	26	22.22
Non-PCI	408	67	16.42
Government Officer	23	22	95.65
Total	592	132	22.30

*PCI* percutaneous coronary intervention, *CR* cardiac rehabilitation, *CCRC* cardiocerebrovascular rehabilitation center

### Differences in organization-wise perception of CR

There were differences in the perceptions among organizations regarding the target diseases (Q4; *p* = 0.010) and the roles of governmental health policies for CR (Q10, 11, 12; *p* = 0.000). In particular, responses to Q11 (roles of governmental health policies for CR) were generally negative among directors of regional cardiocerebrovascular centers (2.50, 2.00–3.75), directors of hospitals that performed PCI but not CR (3.00, 3.00–4.00), and health policy personnel (3.00, 3.00–3.00). The details are shown in Additional file 2.

### Differences in perception of CR based on organizational characteristics

Differences in perception about measures to promote CR between directors of hospitals that did and those that did not perform PCI.

**Fig. 1:**
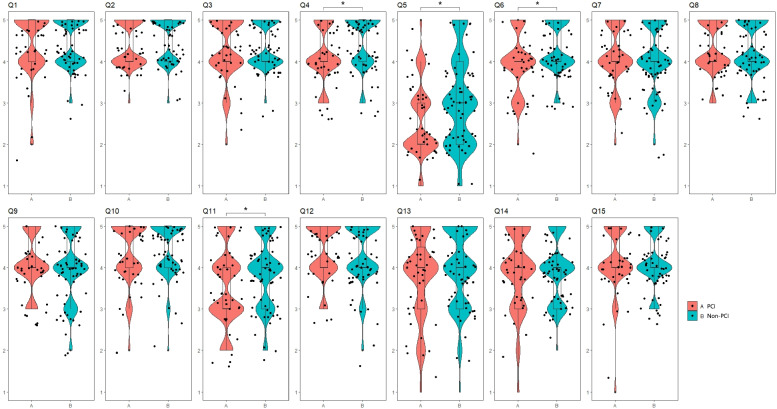
There were significant differences in Q4 (*p* = 0.012), Q5 (*p* = 0.020), Q6 (*p* = 0.031), and Q11 (*p* = 0.037) between PCI and non-PCI

Directors of hospitals that did not perform PCI agreed more to Q4 (target diseases for CR; *p* = 0.012), Q5 (patients’ roles in CR; *p* = 0.020), Q6 (hospitals’ roles in CR; *p* = 0.031), and Q11 (roles of governmental health policies in CR; *p* = 0.037) than those of hospitals that performed PCI. Responses to Q5 (patients’ roles in CR) significantly differed (*p* = 0.020) between directors of hospitals that performed PCI (2.00, 2.00–3.00) and those that did not (3.00, 2.00–4.00; Fig. 1).

Differences in perception about measures to promote CR between directors of hospitals that had and those that did not have a CR system.

**Fig. 2:**
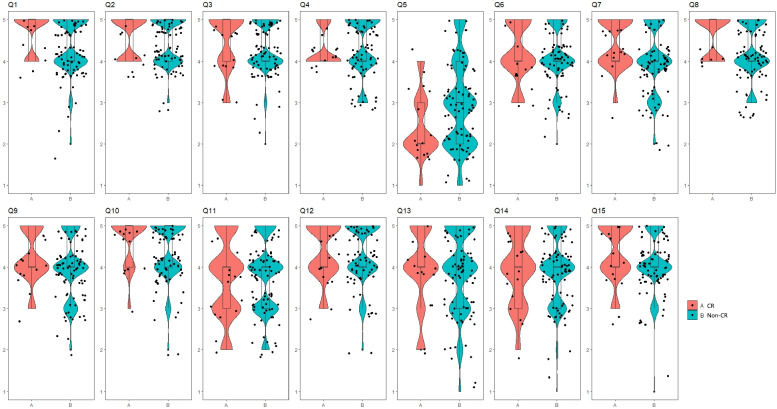
There were positive tendencies for Q1 (*p* = 0.061), Q5 (*p* = 0.055), Q7 (*p* = 0.065), and Q8 (*p* = 0.063) in CR compared with non-CR

Although insignificant, directors of hospitals with CR systems perceived a higher need for Q1 (effectiveness of CR; *p* = 0.061), Q5 (patients’ roles in CR; *p* = 0.055), Q7 (hospitals’ roles in CR; *p* = 0.065), and Q8 (hospitals’ roles in CR; *p* = 0.063) than their counterparts of hospitals without CR systems. Responses to Q5 (patients’ role in CR) indicated a tendency for directors of hospitals without CR systems to place more weight on the patients’ role (3.00, 2.00–4.00) than those of hospitals with a CR system (2.00, 2.00–3.00; Fig. 2).

Differences in perception about measures to promote CR between hospital directors and civil servants.

**Fig. 3:**
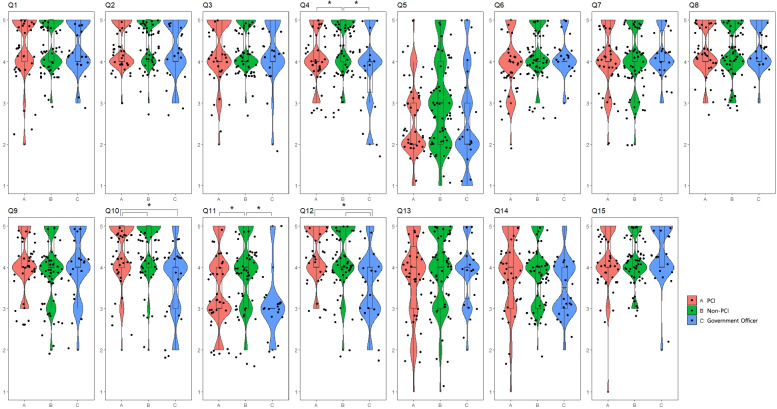
There were significant differences in Q4 (*p* = 0.015), Q10 (*p* = 0.000), Q11 (*p* = 0.003), Q12 (*p* = 0.000), and Q14 (*p* = 0.028) between hospital and government officials

Hospital directors perceived a significantly greater need for Q4 (target diseases for CR; *p* = 0.015), Q10, Q11, Q12, and Q14 (roles of governmental health policies in CR; *p* = 0.000, 0.003, 0.000, and 0.028, respectively) when compared with civil servants. In particular, civil servants indicated low agreement with most statements about the roles of governmental health policies compared with hospital directors. Further, personnel from both hospitals and public organizations indicated negative responses to patients’ roles in promoting CR (Fig. 3).

Differences in perception about measures to promote CR between directors of hospitals that did and those that did not have a CR system.

**Fig. 4:**
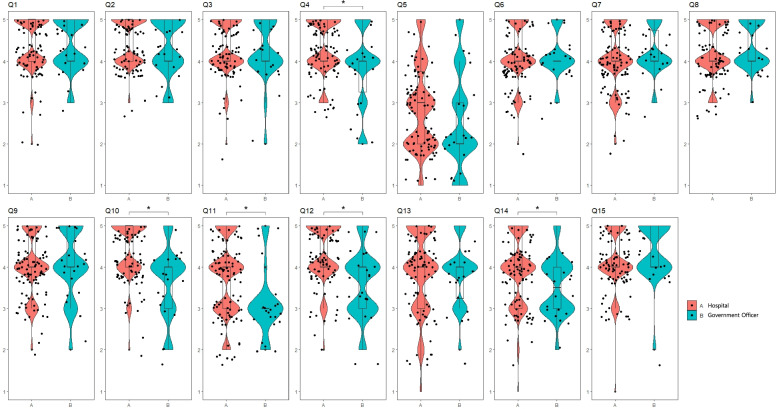
There were significant differences in Q4 (*p* = 0.003), Q5 (*p* = 0.031), Q10 (*p* = 0.000), Q11 (*p* = 0.001), and Q12 (*p* = 0.001) between PCI, non-PCI, and government officials

The responses among three groups were compared using an analysis of variance (ANOVA), and there were significant differences in the responses to Q4 (target diseases for CR; *p* = 0.003), Q5 (patients’ roles in CR; *p* = 0.031), Q10 (roles of governmental health policies in CR; *p* = 0.000), Q11 (roles of governmental health policies in CR; *p* = 0.001), and Q12 (roles of governmental health policies in CR; *p* = 0.001) among the groups. The responses to Q5 significantly differed among the directors of hospitals that performed PCI (2.00, 2.00–3.00), directors of hospitals that did not perform PCI (3.00, 2.00–4.00), and civil servants (2.00, 2.00–3.25; Fig. 4).

Only questions with significantly different responses among the groups in ANOVA were further analyzed with post hoc tests. Responses to Q4 significantly differed between directors of hospitals that did and those that did not perform PCI (*p* = 0.012) and between civil servants and hospitals that did not perform PCI (*p* = 0.003). Responses to Q10 significantly differed between civil servants and hospitals that performed PCI (*p* = 0.001) and civil servants and hospitals that did not perform PCI (*p* = 0.000). Responses to Q11 significantly differed between directors of hospitals that did and those that did not perform PCI (*p* = 0.037) and hospitals that did not perform PCI and civil servants (*p* = 0.000). Responses to Q12 significantly differed between civil servants and hospitals that performed PCI (*p* = 0.001) and civil servants and hospitals that did not perform PCI (*p* = 0.000). The details are shown in Table 2.

**Table 2:** Multiple comparisons test of Kruskal Wallis test of PCI, non-PCI, Government Officer

Dependent Variable	PCI(1) vs. non-PCI(2) vs. Government Officer(3)		N	M(Q1-Q3)	Mean rank	*p*-value
Q4	1–2	1	43	4.00(4.00–5.00)	46.78	.012^*^
		2	67	4.00(5.00–5.00)	61.1	
	1–3	1	43	4.00(4.00–5.00)	34.78	.245
		3	22	3.00(4.00–4.25)	29.52	
	2–3	2	67	4.00(5.00–5.00)	49.29	.003^*^
		3	22	3.00(4.00–4.25)	31.93	
Q5	1–2	1	43	2.00(2.00–3.00)	47.09	.020
		2	67	3.00(2.00–4.00)	60.9	
	1–3	1	43	2.00(2.00–3.00)	33.55	.729
		3	22	2.00(2.00–3.25)	31.93	
	2–3	2	67	3.00(2.00–4.00)	47.85	.060
		3	22	2.00(2.00–3.25)	36.32	
Q10	1–2	1	43	4.00(5.00–5.00)	53.62	.578
		2	67	4.00(5.00–5.00)	56.71	
	1–3	1	43	4.00(5.00–5.00)	38.06	.001^*^
		3	22	3.00(4.00–4.00)	23.11	
	2–3	2	67	4.00(5.00–5.00)	50.71	.000^*^
		3	22	3.00(4.00–4.00)	27.61	
Q11	1–2	1	43	3.00(3.00–4.00)	47.97	.037^*^
		2	67	3.00(4.00–5.00)	60.34	
	1–3	1	43	3.00(3.00–4.00)	35.78	.078
		3	22	3.00(3.00–3.00)	27.57	
	2–3	2	67	3.00(4.00–5.00)	50.26	.000^*^
		3	22	3.00(3.00–3.00)	28.98	
Q12	1–2	1	43	4.00(4.00–5.00)	56.43	.787
		2	67	4.00(4.00–5.00)	54.9	
	1–3	1	43	4.00(4.00–5.00)	38.44	.001^*^
		3	22	3.00(4.00–4.00)	22.36	
	2–3	2	67	4.00(4.00–5.00)	50.11	.000^*^
		3	22	3.00(4.00–4.00)	29.43	

*PCI* percutaneous coronary intervention; *, *p* < 0.017 by Mann Whitney U test

## Discussion

This study investigated the discrepancies in the perceptions of the need for CR programs and health policies among directors of four types of healthcare facilities (hospitals that performed PCI and CR, those that performed PCI but not CR, those that did not perform PCI, and regional cardiocerebrovascular centers) and health policy directors in public agencies. We sent the questionnaire to the directors of each hospital and health policy personnel and requested a response, and 22.30% of them responded with a completed questionnaire. The participants generally agreed with statements about CR being effective on patients with other CVDs or chronic conditions, the need for policies to promote CR and provide financial support, and the government’s role in offering incentives to patients or healthcare facilities to boost participation in CR. However, the directors of regional cardiocerebrovascular centers, directors of hospitals that performed PCI but not CR, and health policy personnel generally showed low agreement.

The participants were reclassified according to their organizations’ characteristics for further analysis. Directors of hospitals that did not perform PCI (compared with those of hospitals that did), hospital directors (compared with civil servants), and directors of hospitals that did not perform PCI (compared with those of hospitals that do and directors of public agencies) believe that CR is effective for patients with other CVDs or chronic conditions (Q4). Directors of hospitals that did not perform PCI (compared with those of hospitals that did) and directors of hospitals that do not perform PCI (compared with those of hospitals that do and directors of public agencies) thought that patients are responsible for the management of heart disease-related risk factors (Q5).

Compared with directors of hospitals that performed PCI, those of hospitals that did not perform PCI perceived hospitals as playing an important role in identifying and managing the barriers to CR participation (Q6). Some of the universal barriers to CR pertinent to the hospital system included lack of expertise among professionals, lack of resources (e.g., time, staff, facilities, and equipment), and awareness, attitude, and safety problems [14]. Grace et al. [15] emphasized the significance of CR referral timing, provision of CR-related information to patients at discharge, and the roles of cardiologists, internal medicine specialists, family doctors, nurses, and relevant experts (building trust with patients and promoting and recommending CR). Further, many healthcare managers stated in interviews that CR programs need to be implemented in hospitals and communities [16].

Compared with civil servants, hospital directors perceived the government as playing an important role in promoting CR in terms of implementing policies and providing financial support (Q10). An earlier study revealed that directors of facilities with established CR systems agreed more than those without CR systems that more funding is required to support CR programs [16]. Healthcare managers generally perceived that health authorities inadequately fund CR services and that many policymakers are hesitant about allocating a budget for disease prevention among the public. In fact, CR is mostly funded by public funds, but patients still pay more than half the average cost of treatment [17]. On the other hand, governmental policy stakeholders believed that there is little evidence to justify CR’s cost-effectiveness and that budget allocation requires more caution because providing public funds for modifying individual lifestyles can infringe upon personal freedoms [15]. One common finding between previous studies [15, 16] and the current study is that policymakers are hesitant about funding CR services. This is in line with the results of a South Korean study [18] that found that CR programs cannot be promoted solely by healthcare facilities and require governmental policies and financial support.

In this study, directors of hospitals that did not perform PCI (compared with those of hospitals that did), hospital directors (compared with civil servants), and directors of hospitals that did not perform PCI (compared with those of hospitals that did and directors of public agencies) believed that government-offered patient incentives are important to promote CR (Q12). Further, they perceived the government as playing an important role in guaranteeing the time and right to participate in CR (Q14). According to the American Association of Cardiovascular and Pulmonary Rehabilitation, 33% of hospitals offer incentives, and 16% provide CR therapies in the evening to boost participation in CR [19]. Batalik et al. showed the potentially positive effect on increasing CR participation with remotely monitored telerehabilitation for CR as alternative methods for driving CR participation [20].

This study’s findings also revealed that hospital directors generally perceive the need for such types of support. The directors of hospitals with CR systems tended to more positively perceive CR’s effectiveness in facilitating lifestyle changes in patients (Q1) and perceived hospitals as playing an important role in retaining CR personnel and the relevant systems and providing information to promote CR (Q 7, 8) when compared with directors of hospitals without CR systems. Implementing CR systems appears to be associated with a positive perception of CR.

There have been several efforts to increase CR use around globe such as Million Hearts Program [21] and European Alliance for Cardiovascular Health (EACH) Cardiovascular Health (CVH) Plan in medically advanced countries [22]. In future, in accordance with these movement, we should prepare the strategic plan to increase CR participation in Asian country.

One key benefit of this study is that it simultaneously compared and analyzed the opinions of hospital directors and policy personnel in public agencies and made use of nationwide data. However, since the actual response rate was very low and the study was only conducted in South Korea, the findings need to be interpreted with caution.

## Conclusion

Hospital and health policy directors generally exhibited neutral to positive perceptions about factors related to CR promotion. However, each factor’s perceived contribution varied across organizations. Notably, the perceived importance of governmental policy-related factors was slightly lower among directors of hospitals without established CR systems and those of health policy agencies. As suggested by our study, governmental socioeconomic support for CR, such as providing incentives, implementing night-time CR therapies, and linking hospital programs to community-based programs, is crucial, along with hospitals’ roles in timely CR referral and patient education about the need for CR.

## Supplementary Information

**Additional file 1.**

**Additional file 2.**

## Data Availability

Datasets are available from the corresponding author on reasonable request.

## Abbreviations

ANOVA: Analysis of variance
CR: Cardiac rehabilitation
CVD: Cardiovascular disease
PCI: Percutaneous coronary intervention
