# Supplementary material for: A survey of the perceptions of barriers to and facilitators of cardiac rehabilitation in healthcare providers and policy stakeholders
Source: BMC Health Serv Res. 2022 Aug 5;22:999. doi: 10.1186/s12913-022-08298-3 (PMC9356496; doi:10.1186/s12913-022-08298-3)
Supplement: Supplementary file 1 — Additional file 1. [file 12913_2022_8298_MOESM1_ESM.docx]

Additional file 1

**CR questionnaire for hospital managers/policy personnel**

After receiving acute-phase treatment for cardiovascular disease (CVD), patients still suffer from vascular arteriosclerosis that continues to progress, increasing their risk for recurrence and premature death. To lower such a risk, patients need to undergo hospital-based cardiac rehabilitation (CR) in the outpatient setting for approximately 3 months following discharge, which should be followed by community-based CR. Community-based CR involves patients’ practice of abstinence from tobacco, proper diet, weight control, and adequate exercise and physical activity at home, and in developed countries, community organizations (community healthcare facilities or non-healthcare facilities) undertake the role of managing and supervising such that patients can comply with these practices. South Korea lacks such a system, and it is essential to develop relevant measures and strategies.

This project is a national policy project commissioned by the Department of Cardiovascular Disease at the Korea Disease Control and Prevention Agency of the Ministry of Health and Welfare. This survey aims to determine whether resources in various healthcare and non-healthcare facilities in communities can be utilized for community-based CR. We will foster an effective environment for community-based CR based on the findings.


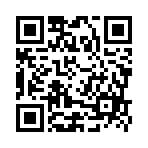


**Response number_____________**

This survey is conducted electronically.

If you choose to use a cellphone, please scan the QR code, and it will direct you to the online questionnaire.

If you choose to use a computer, go to [**www.crsurvey.co.kr**](http://www.crsurvey.co.kr) and click on **6_Questionnaire for hospital managers/policy personnel** to be directed to the online questionnaire.

Please remember to write the response number at the top corner before answering the questionnaire. This number is only used for preventing multiple questionnaire participation. After you finish the questionnaire, you will see instructions about receiving a mobile coupon as a token of appreciation for your participation. If you wish to submit the questionnaire via mail, please request a return envelope from Ji-Sun Heo ([sun440@naver.com](mailto:sun440@naver.com) 010-8310-7327). The deadline for questionnaire submission is **July 3 (Friday).**

Thank you.

**Please choose () the most appropriate response for each statement below.**

|  | **Strongly disagree** | **Disagree** | **Neutral** | **Agree** | **Strongly agree** |
| --- | --- | --- | --- | --- | --- |
| 1. CR facilitates changes in patients’ behavior and habits to improve their cardiovascular health. |  |  |  |  |  |
| 1. CR programs contribute to recovering cardiopulmonary endurance, improving risk factors, and enhancing patients’ quality of life. |  |  |  |  |  |
| 1. CR programs contribute to lowering patients’ readmission and mortality rates after discharge. |  |  |  |  |  |
| 1. CR is necessary even for patients with other CVDs, such as stroke or other chronic conditions, including diabetes. |  |  |  |  |  |
| 1. Patients with CVD and their families are fully responsible for their behavior-habit modification and risk factor management. |  |  |  |  |  |
| 1. Hospitals must identify barriers to CR and devise strategies to overcome them. |  |  |  |  |  |
| 1. Hospitals should be equipped with CR personnel and system to enable a systematic CR referral. |  |  |  |  |  |
| 1. Hospitals must provide information about CR to patients such that patients can undergo CR. |  |  |  |  |  |
| 1. If I have the power to make the decision, I will provide the facilities, equipment, and personnel required for CR programs. |  |  |  |  |  |
| 1. The government must not only establish policies and systems to promote CR but also provide unreserved financial support. |  |  |  |  |  |
| 1. The government must offer incentives of any form to patients participating in CR to promote CR. |  |  |  |  |  |
| 1. The government must offer incentives of any form to healthcare facilities that provide CR to promote CR. |  |  |  |  |  |
| 13. Patients’ out-of-pocket costs for CR should be lowered to increase their participation in CR (apply benefit extension). |  |  |  |  |  |
| 14. Patients who require CR should be guaranteed the right (time) to participate in outpatient CR (especially working individuals). |  |  |  |  |  |
| 15. A community-based CR system should be established such that patients can continue receiving care after hospital-based CR. |  |  |  |  |  |
| 16. Other opinions about CR programs: | | | | | |
