# Supplementary material for: A survey of the perceptions of barriers to and facilitators of cardiac rehabilitation in healthcare providers and policy stakeholders
Source: BMC Health Serv Res. 2022 Aug 5;22:999. doi: 10.1186/s12913-022-08298-3 (PMC9356496; doi:10.1186/s12913-022-08298-3)
Supplement: Supplementary file 2 — Additional file 2. [file 12913_2022_8298_MOESM2_ESM.docx]

Supplementary Table. 1 Comparison of reply for each survey item according to characteristics.

|  | PCI, CR  (n=13) | CCRC  (n=4) | PCI, Non-CR (n=26) | Non-PCI  (n=67) | Government Officer  (n=22) | p |
| --- | --- | --- | --- | --- | --- | --- |
| Q1 | 5.00(4.50-5.00) | 4.50(4.00-5.00) | 4.00(4.00-5.00) | 5.00(4.00-5.00) | 4.00(4.00-5.00) | 0.160 |
| Q2 | 5.00(4.00-5.00) | 4.50(4.00-5.00) | 4.00(4.00-5.00) | 5.00(4.00-5.00) | 4.00(4.00-5.00) | 0.207 |
| Q3 | 5.00(4.00-5.00) | 4.50(3.25-5.00) | 4.00(4.00-5.00) | 4.00(4.00-5.00) | 4.00(4.00-5.00) | 0.534 |
| Q4 | 4.00(4.00-4.50) | 4.50(4.00-5.00) | 4.00(3.00-5.00) | 5.00(4.00-5.00) | 4.00(3.00-4.25) | 0.010^*^ |
| Q5 | 2.00(2.00-3.00) | 2.00(2.00-2.75) | 2.50(2.00-3.00) | 3.00(2.00-4.00) | 2.00(2.00-3.25) | 0.103 |
| Q6 | 4.00(4.00-5.00) | 4.00(3.25-4.75) | 4.00(3.00-4.00) | 4.00(4.00-5.00) | 4.00(4.00-4.25) | 0.106 |
| Q7 | 5.00(4.00-5.00) | 4.00(3.25-4.75) | 4.00(3.00-4.00) | 4.00(4.00-5.00) | 4.00(4.00-5.00) | 0.134 |
| Q8 | 5.00(4.00-5.00) | 4.50(4.00-5.00) | 4.00(4.00-5.00) | 4.00(4.00-5.00) | 4.00(4.00-5.00) | 0.331 |
| Q9 | 4.00(4.00-5.00) | 4.00(3.25-4.75) | 4.00(3.75-4.25) | 4.00(4.00-5.00) | 4.00(3.75-5.00) | 0.774 |
| Q10 | 5.00(5.00-5.00) | 4.00(3.25-4.75) | 4.00(4.00-5.00) | 5.00(4.00-5.00) | 4.00(3.00-4.00) | 0.000^*^ |
| Q11 | 4.00(3.00-5.00) | 2.50(2.00-3.75) | 3.00(3.00-4.00) | 4.00(3.00-5.00) | 3.00(3.00-3.00) | 0.001^*^ |
| Q12 | 5.00(4.00-5.00) | 4.00(3.25-4.00) | 4.50(4.00-5.00) | 4.00(4.00-5.00) | 4.00(3.00-4.00) | 0.001^*^ |
| Q13 | 4.00(3.50-5.00) | 4.00(2.50-4.75) | 4.00(3.00-4.25) | 4.00(3.00-5.00) | 4.00(3.00-4.00) | 0.784 |
| Q14 | 4.00(3.00-5.00) | 4.00(4.00-4.75) | 4.00(3.00-5.00) | 4.00(3.00-4.00) | 3.50(3.00-4.00) | 0.226 |
| Q15 | 5.00(4.00-5.00) | 4.00(4.00-4.75) | 4.00(4.00-5.00) | 4.00(4.00-5.00) | 4.00(4.00-5.00) | 0.463 |

PCI, percutaneous coronary intervention; CR, cardiac rehabilitation; CCRC, cardiocerebrovascular rehabilitation center; Values describe as median (interquartile range)
